# Supplementary material for: Deciphering the Coevolutionary Dynamics of L2 β-Lactamases via Deep Learning
Source: J Chem Inf Model. 2024 Apr 30;64(9):3706–17. doi: 10.1021/acs.jcim.4c00189 (PMC11094718; doi:10.1021/acs.jcim.4c00189)
Supplement: Supplementary file 1 — ci4c00189_si_001.pdf [file ci4c00189_si_001.pdf]

Supplementary Information

Deciphering the co-evolutionary dynamics of L2 β-lactamases via Deep learning

Yu Zhu, Jing Gu, Zhuoran Zhao, A W Edith Chan, Maria F. Mojica,

Andrea M. Hujer, Robert A. Bonomo, Shozeb Haider

Table S1. Feature selections for the six class A β-lactamase.

System	Numbering Reference	Hydrophobic Nodes	Binding Site Residues
**KPC-2**	PDB ID: 3DW0	G74, F75, L76, A77, A78, A79, V80, L81, A82, A101, L102, V103, S106, P107, I108, A124, A125, A126, V127, A133, A134, A135, L137, L138, L139, A185, V186, T187, A198, L199, A200, A201, A223, A224, V225, A230, V231, G232, A248, V249, V250, V260, L261, A262, A280, A281, A282, A283	S70, K73, P104, W105, P107, S130, N132, E166, L167, N170, T216, K234, T235, G236, T237, C238, G239
**SME-1**	PDB ID: 1DY6	G74, F75, L76, A77, A78, A79, V80, L81, E82, D101, L102, E103, S106, P107, I108, S124, A125, A126, L127, G133, A134, T135, I137, I138, M139, A185, V186, A187, V198, L199, N200, A201, A223, S224, V225, V230, V231, G232, A248, V249, I250, I260, V261, S262, A280, E281, A282, S283	S70, K73, Y104, H105, P107, S130, N132, E166, L167, N170, T216, K234, T235, G236, S237, C238, G239
**L2a**	UniProt ID: P96465	S87, V88, L89, A90, A91, T92, V93, L94, S95, D114, L115, L116, A119, P120, V121, R137, A138, T139, I140, T146, A147, A148, L150, L151, F152, A198, M199, A200, V211, L212, Q213, L214, A236, G237, L238, R243, V244, R245, A260, V261, L262, V282, L283, T284, A292, Q293, V294, G295	S83, K86, S117, H118, P120, S143, N145, E179, P180, N183, T229, K247, T248, G249, S250, N251, G252
**L2b**	PDB ID: 5NE2	S87, M88, L89, A90, A91, T92, V93, L94, S95, D114, L115, L116, A119, P120, V121, R137, A138, T139, I140, T146, A147, A148, L150, L151, F152, A198, M199, A200, V211, L212, Q213, P214, A236, G237, L238, R243, V244, G245, A260, V261, L262, V282, L283, T284, A292, Q293, V294, G295	S83, K86, S117, H118, P120, S143, N145, E179, P180, N183, T229, K247, T248, G249, S250, N251, G252
**L2c**	UniProt ID: P96465	S87, V88, L89, A90, A91, T92, V93, L94, S95, D114, L115, L116, A119, P120, V121, R137, A138, T139, I140, T146, A147, A148, L150, L151, F152, A198, M199, A200, V211, L212, Q213, P214, A236, G237, L238, R243, V244, G245, A260, V261, L262, V282, L283, T284, A292, Q293, V294, G295	S83, K86, S117, H118, P120, S143, N145, E179, P180, N183, T229, K247, T248, G249, S250, N251, G252
**L2d**	UniProt ID: P96465	S88, M89, L90, V91, A92, H93, V94, L95, S96, D115, L116, L117, A120, P121, V122, R138, G139, T140, L141, T147, A148, A149, L151, L152, L153, A199, M200, A201, G212, L213, Q214, F215, A237, G238, L239, R244, V245, G246, A261, V262, L263, V283, V284, T285, A293, R294, V295, G296	S84, K87, S118, Y119, P121, S144, N146, E180, P181, N184, T230, K248, T249, G250, S251, N252, G253

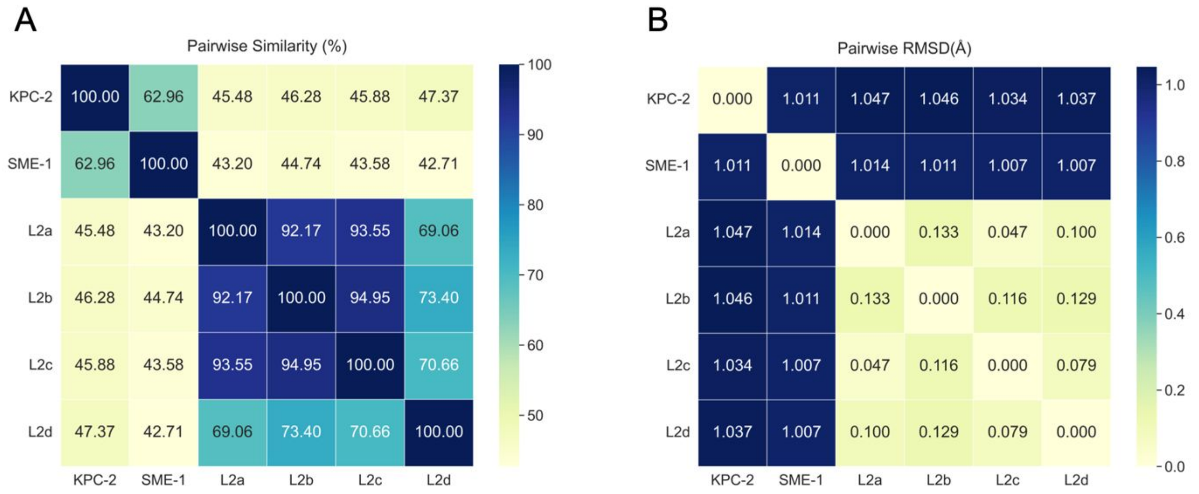

Figure S1. Sequence and structure alignments. (A) The pairwise sequence similarity of the six class A β-lactamases. (B) The pairwise structural Cα RMSD of the six systems.

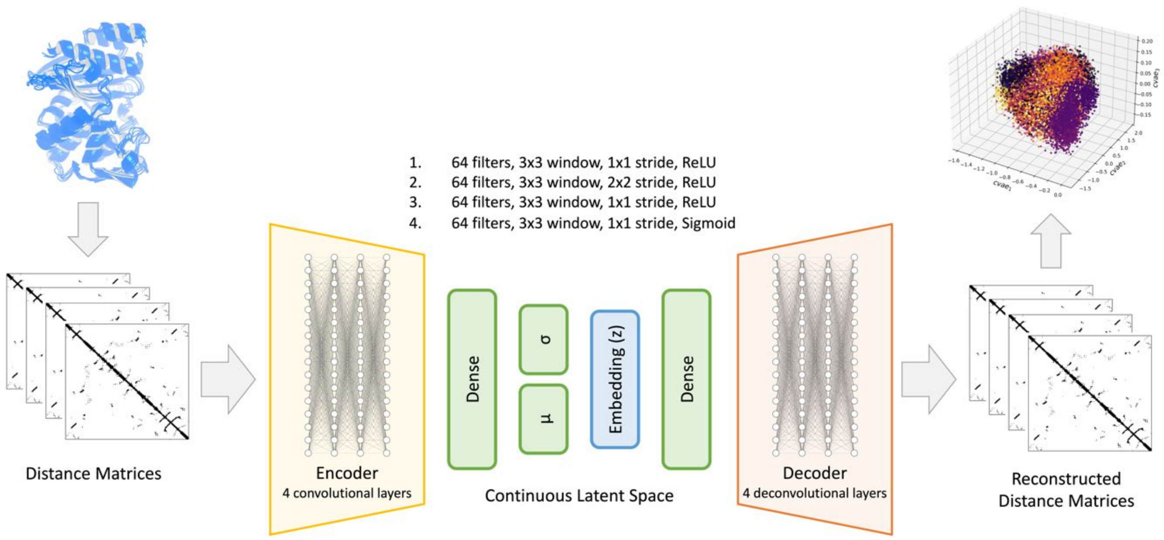

Figure S2. Core architecture of the CVAE-based deep learning approach. Convolutional Variational Autoencoder (CVAE) is a type of artificial neural network that employs the principles of both Convolutional Neural Networks (CNNs) and Variational Autoencoders (VAEs). It uses convolutional networks as its encoder and decoder and leverages the principles of variational inference to learn a structured representation of the input, enabling the generation of new, similar data. Simulation trajectories and feature selections are required for the model training. The decoded embeddings can be processed via diverse dimension reducing methods. The CVAE provides a holistic view of protein structures and their dynamic changes, enabling more precise clustering of different protein conformations. This ability to capture and cluster subtly different protein conformations has applications across various areas, from advancing our understanding of diseases linked to protein misfolding to facilitating drug discovery by mapping potential drug-binding pockets in different conformational states.

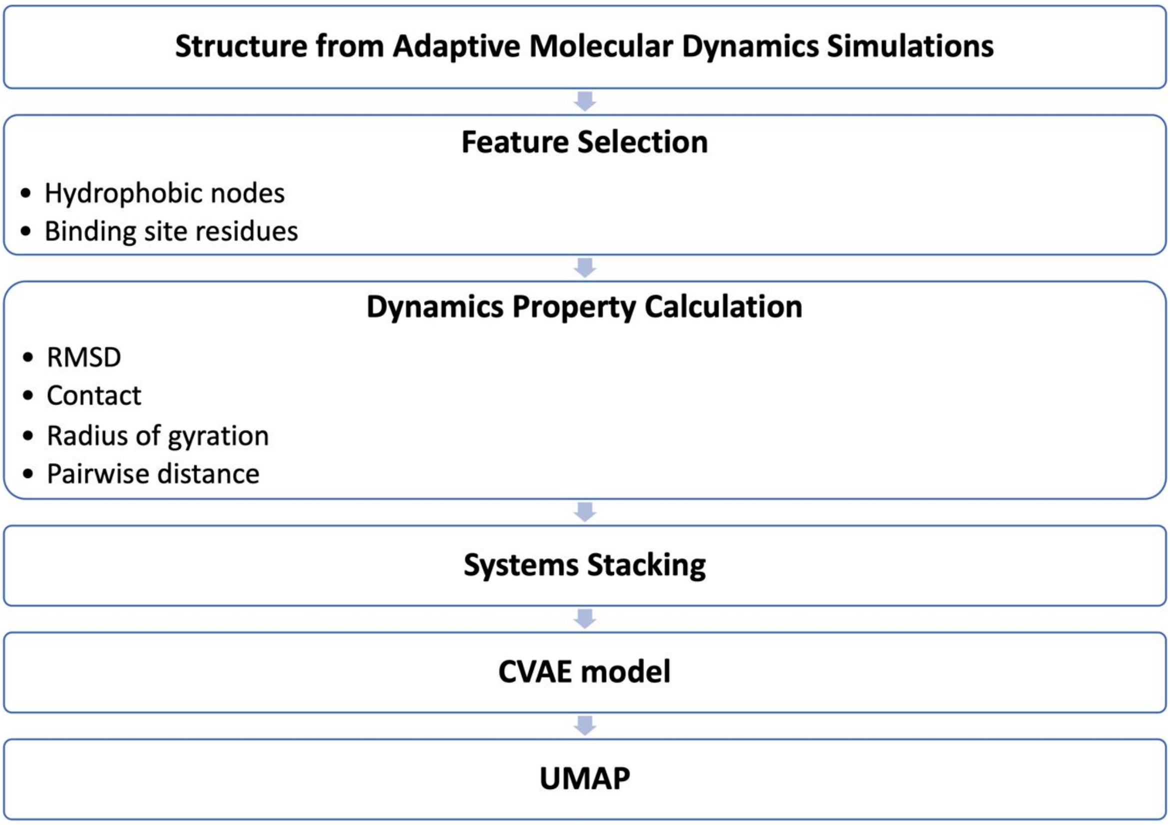

Figure S3. Flowchart of CVAE based clustering.

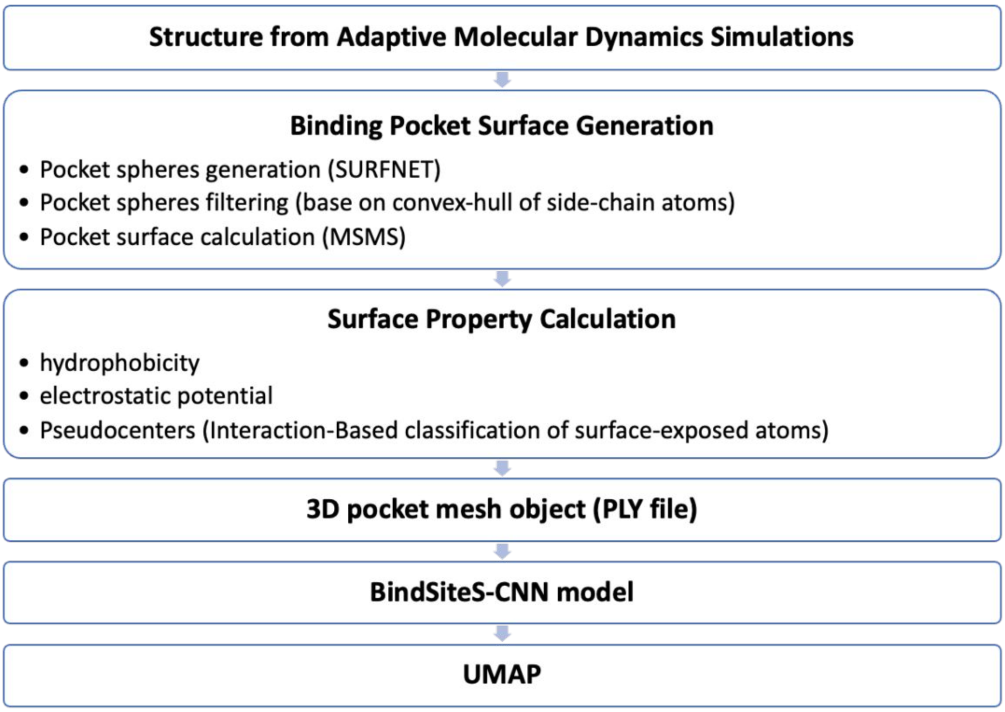

Figure S4. Flowchart of BindSiteS-CNN based binding site comparison.

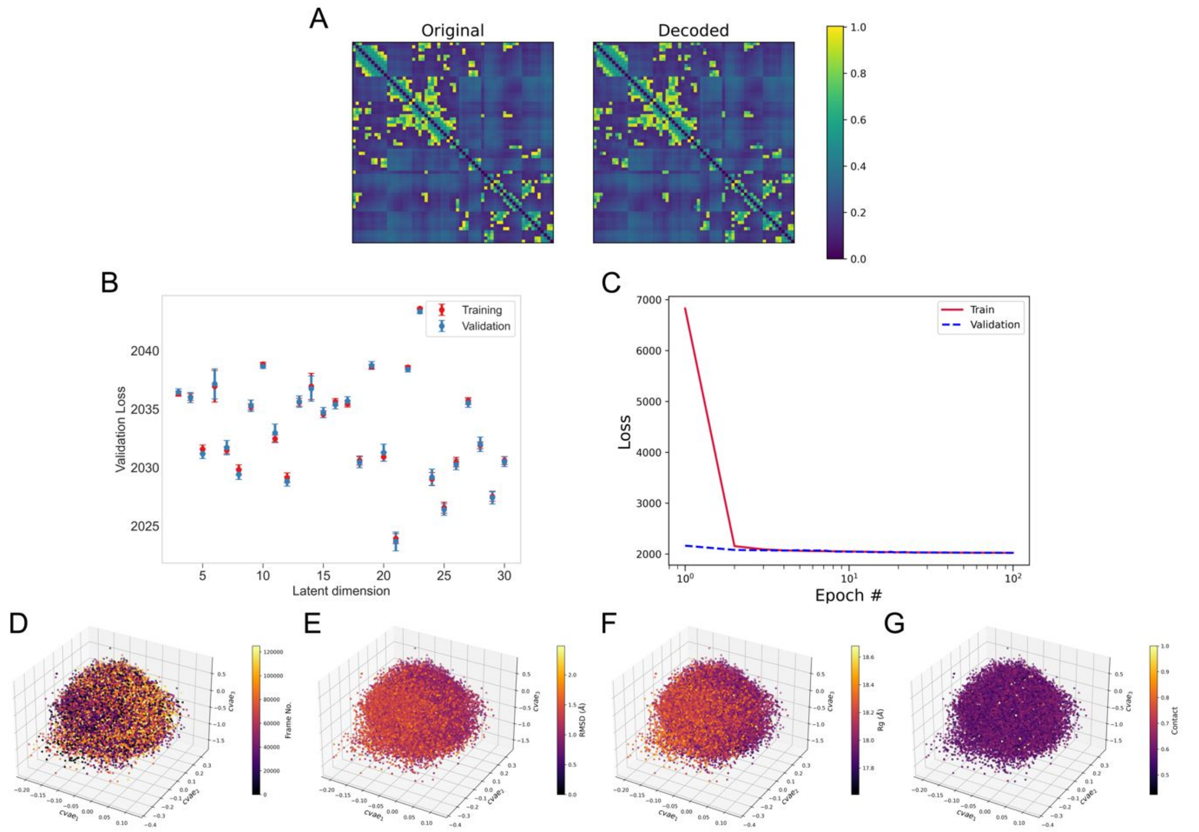

Figure S5. CVAE performance using the four L2 β-lactamases trajectories. (A) Comparison between the original distance matrix and the decoded matrix. (B) Loss for each latent dimension. (C) Model training and validation for the 21st latent space dimension for each epoch. (D), (E), (F) and (G) represent the 3D embeddings of the stacked systems in frame number, RMSD, radius of gyration, contact, respectively.

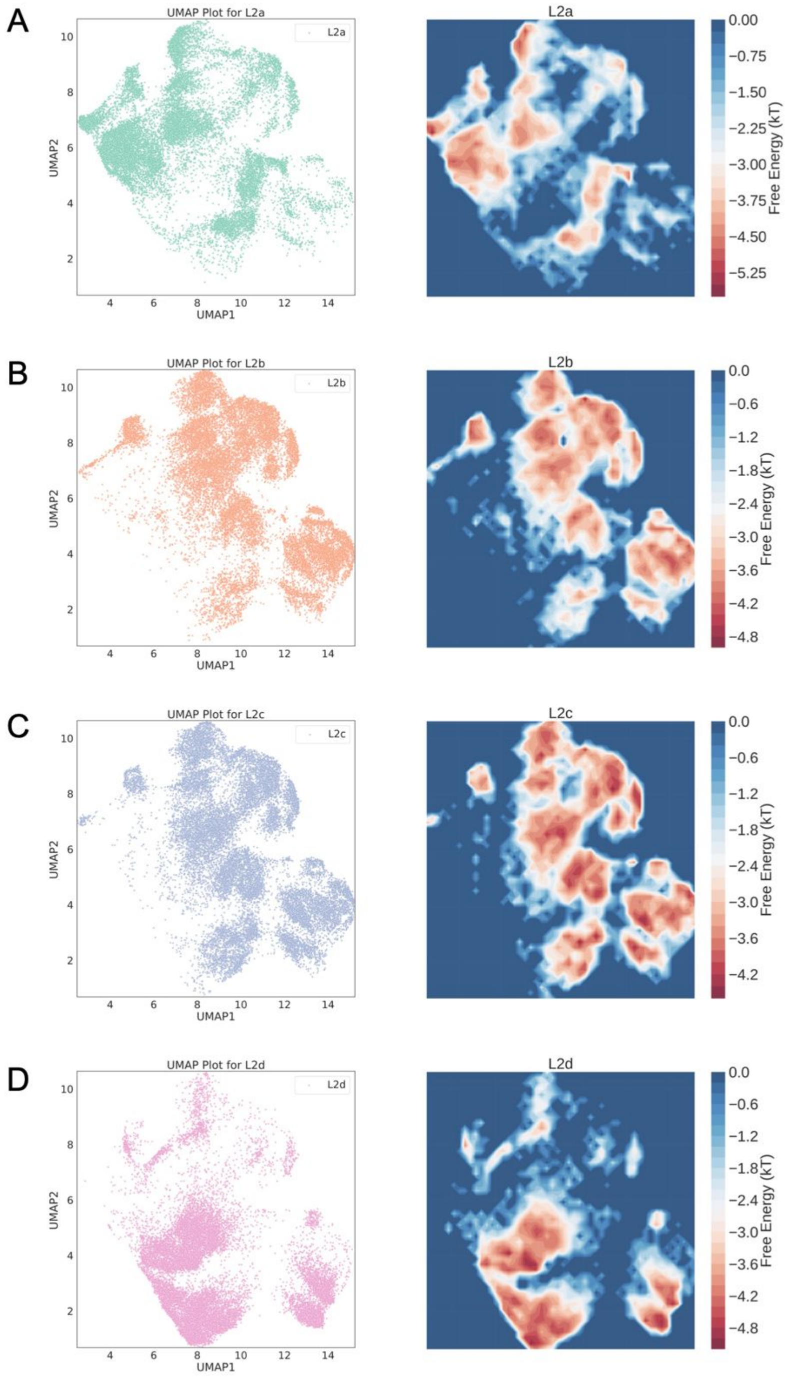

Figure S6. Separation of the 2D UMAP projections of the L2 β-lactamase embeddings. From (A) to (D) were the distinct dynamic clusters and the corresponding free energy landscape of L2a (green), L2b (orange), L2c (amethyst), L2d (violet) systems, respectively.

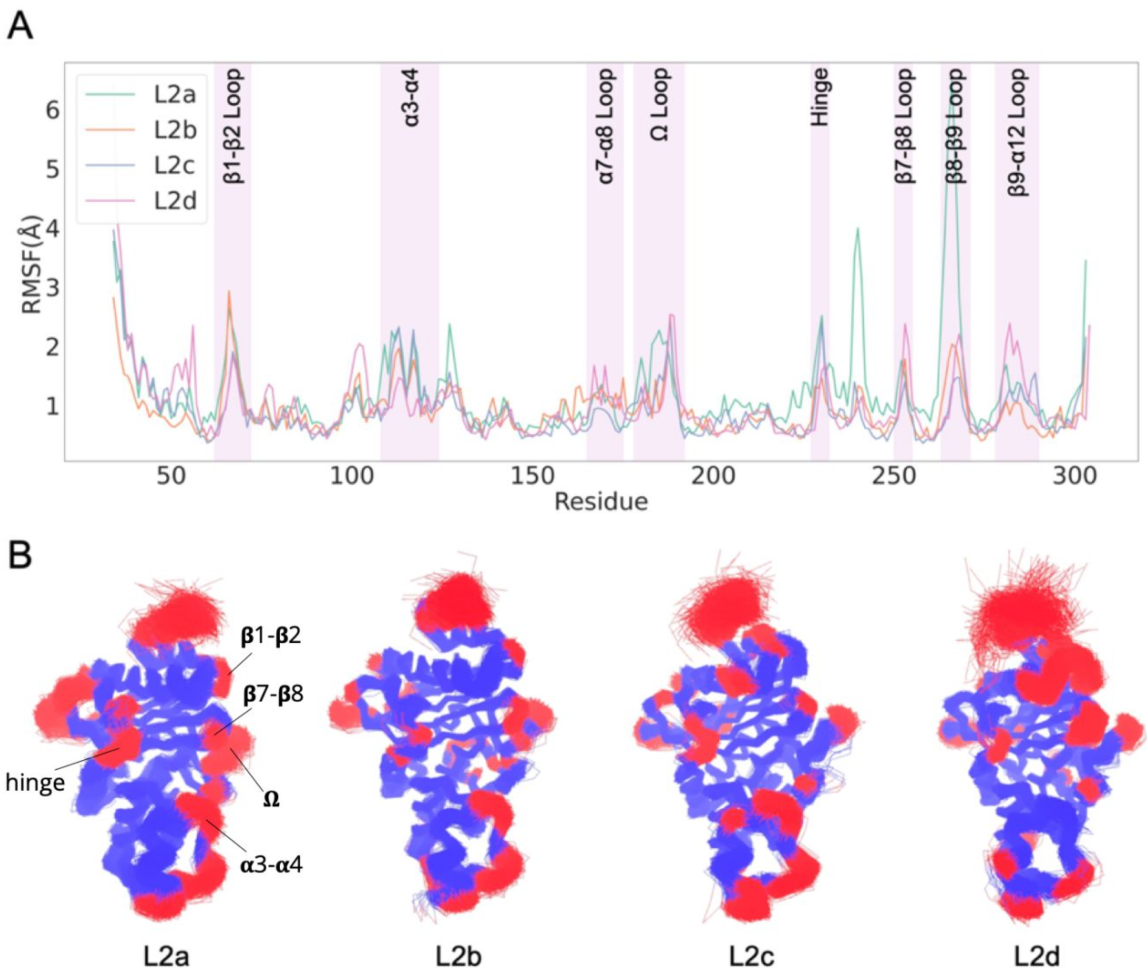

Figure S7. Flexibility of the L2 β-lactamases. (A) Root-mean-square-fluctuation (RMSF) curves of the L2a (green), L2b (orange), L2c (amethyst) and L2d (violet). Key regions were highlighted with purple boxes and secondary structure annotations. (B) From left to right are the dynamical characterisations of four different systems, L2a, L2b, L2c, and L2d β-lactamase, respectively. The colours describe a relative stable core (blue) and flexible surrounding loops (red).
